# Supplementary material for: SARS-CoV-2 Spike triggers barrier dysfunction and vascular leak via integrins and TGF-β signaling
Source: Nat Commun. 2022 Dec 9;13:7630. doi: 10.1038/s41467-022-34910-5 (PMC9734751; doi:10.1038/s41467-022-34910-5)
Supplement: Supplementary file 1 — Supplementary Information [file 41467_2022_34910_MOESM1_ESM.pdf]

Supplementary Information

Title: SARS-CoV-2 Spike triggers barrier dysfunction and vascular leak via integrins and TGF-β signaling

Inventory of Supporting Information

Supplementary Figures:

Supplementary Figure 1

Supplementary Figure 2

Supplementary Figure 3

Supplementary Figure 4

Supplementary Figure 5

Supplementary Figure 6

Supplementary Figure 7

Supplementary Tables:

Supplementary Table 1

Supplementary Table 2

Supplementary Table 3

Supplementary Figures

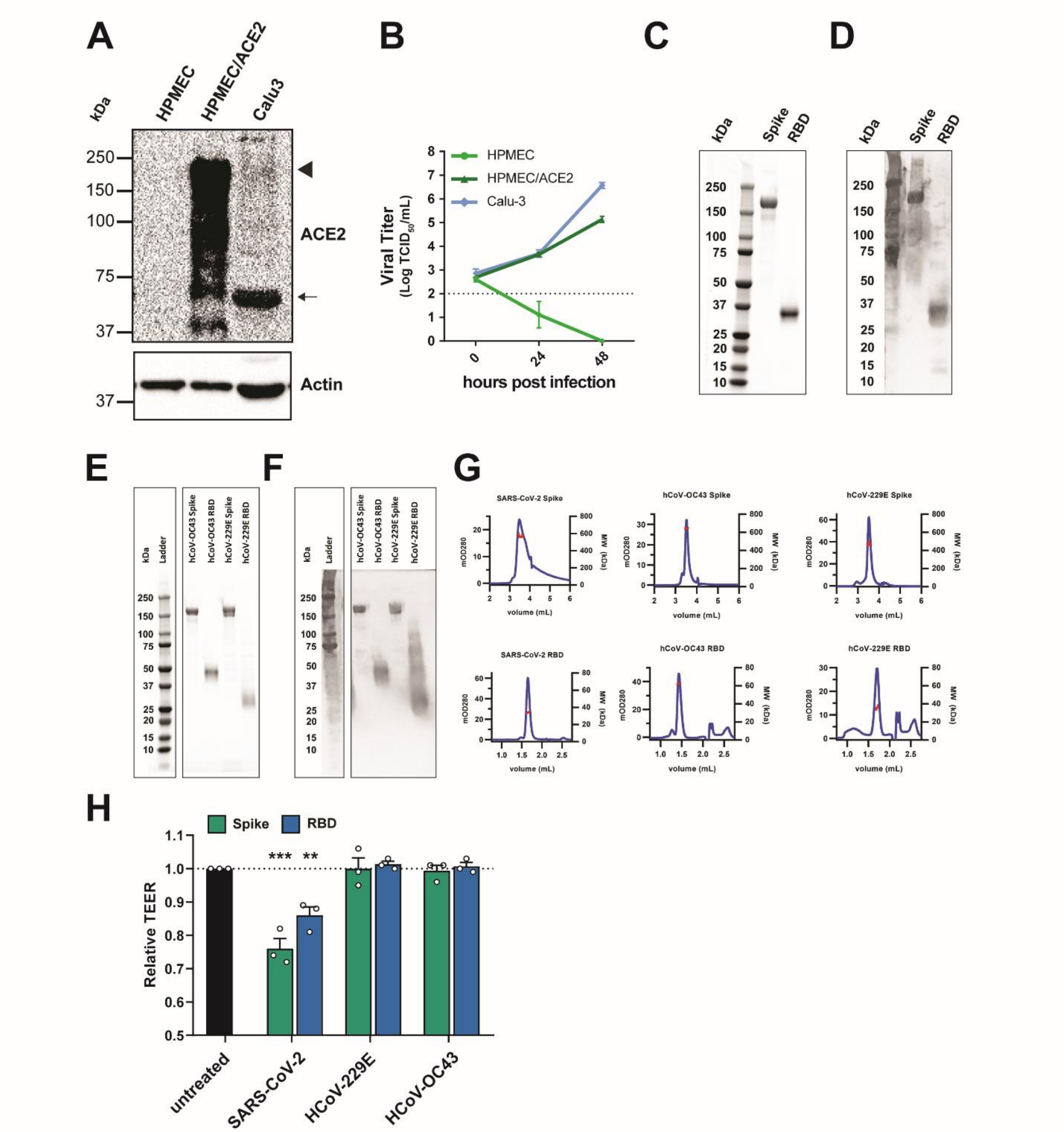

Figure S1. In-house produced coronavirus S is pure but only SARS-CoV-2 S mediates barrier dysfunction (Related to Figure 1). (A) Western blot analysis of ACE2 expression in HPMECs, HPMEC/ACE2, and Calu-3 cells. The arrowhead points to the predicted size of ACE2, while the arrow points to a smaller truncated band. Actin was used as a loading control. Shown is one representative experiment from n=3 biological replicates. **(B)** Growth curve of HPMECs, HPMEC/ACE2, and Calu-3 cells infected with SARS-CoV-2 at an MOI of 0.005, with infectious virus quantified by $TCID_{50}$ at the indicated time-points. Data displayed are from n=3 biological replicates. The dotted line is the limit of detection (LOD) of the assay. **(C and E)** Western blot analysis of home-made full-length trimeric spike and RBD detected by an anti-6xHIS antibody for

SARS-CoV-2 in C and HCoV-229E/HCoV-OC43 in E. **(D and F)** SDS-PAGE visualized by silver stain of home-made full-length trimeric spike and RBD for SARS-CoV-2 in D and HCoV-229E/HCoV-OC43 in F. **(G)** Size-exclusion chromatography of in-house-produced full-length trimeric spike (top) and RBD (bottom), as indicated. **(H)** A TEER assay measuring the barrier of monolayers of HPMECs 24 hours after the indicated coronavirus S treatments at 10 µg/mL. Dotted line is the normalized untreated control condition. Data are from n=3 biological replicates. All data are plotted as mean +/- SEM, with $^{**}p<0.01$ and $^{***}p<0.001$ by One-Way ANOVA with Tukey's Multiple comparisons test compared to untreated controls. Source data are provided as a Source Data file.

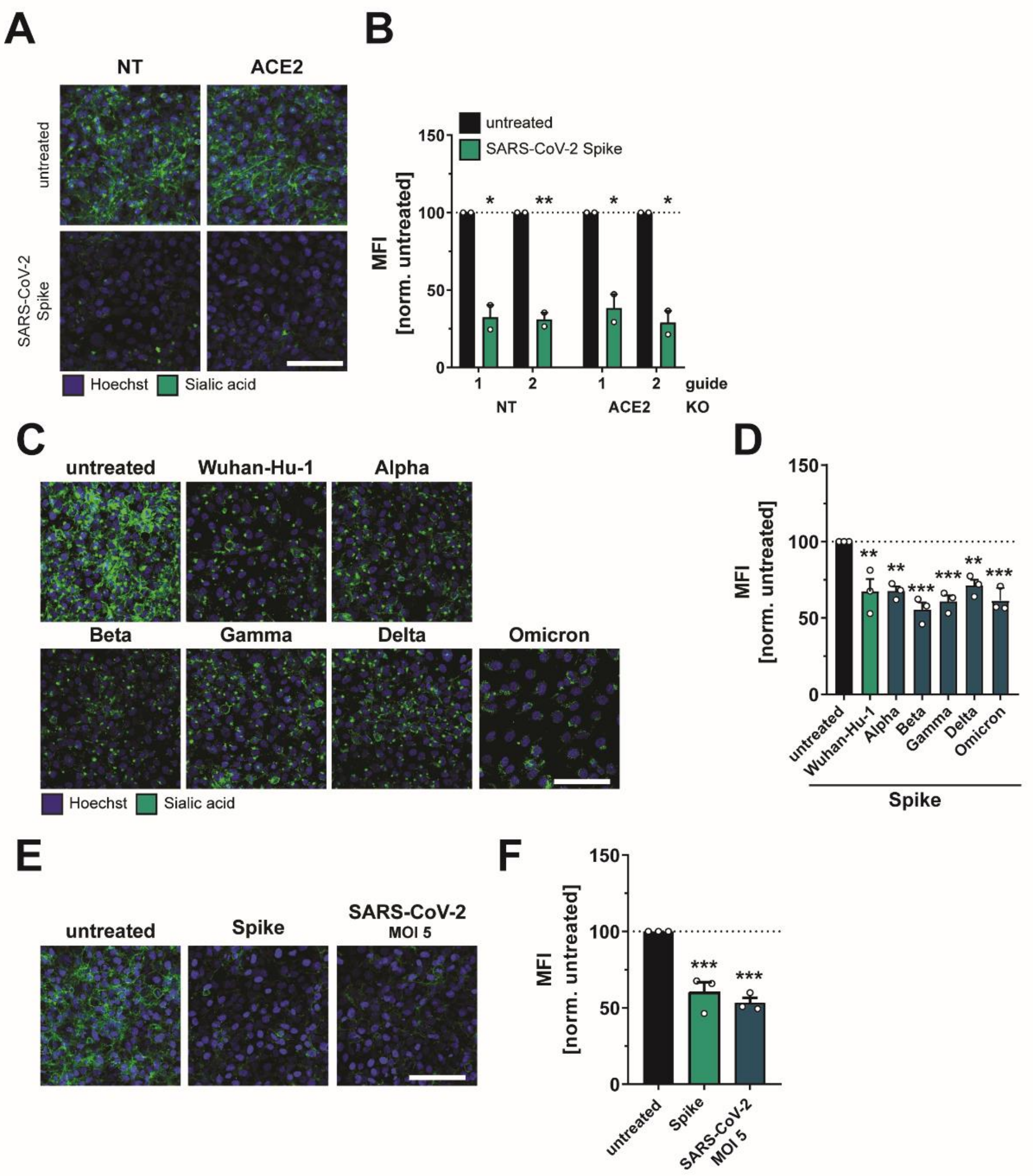

Figure S2. SARS-CoV-2 S from multiple variants mediate barrier dysfunction in an ACE2-independent manner (Related to Figure 2). **(A)** Sialic acid EGL assay on HPMEC transduced with lentivirus-encoding guide RNAs targeting the indicated genes, treated with 10 µg/mL of S and imaged at 24 hpt. Shown are representative images from n=2 biological replicates. **(B)** Quantification of A from n=2 biological replicates; Control guide data from this panel are from the same experiment as Figure 4F. **(C)** Sialic acid EGL disruption assay of HPMECs treated with SARS-CoV-2 S (10 µg/mL) from the indicated variants imaged at 24 hpt. **(D)** Quantification of C from n=3 biological replicates. **(E)** Sialic acid EGL disruption assay of HPMEC treated with SARS-CoV-2 S (10 µg/mL) or inoculated with SARS-CoV-2 WA/1 at an MOI of 5, imaged at 24 hpt. **(F)** Quantification of E from n=3 biological replicates. For all panels, sialic acid is stained in green and nuclei are stained with Hoechst in blue with scale bars at 50 µm. MFI is mean fluorescence intensity. Dotted lines are the normalized untreated control conditions. All data are plotted as

mean +/- SEM with *$p<0.05$, **$p<0.01$, ***$p<0.001$, and n.s. $p>0.05$ by One-Way ANOVA with Tukey's Multiple comparisons test except for (B) which was analyzed by two-sided unpaired t-test compared to untreated controls. Source data are provided as a Source Data file.

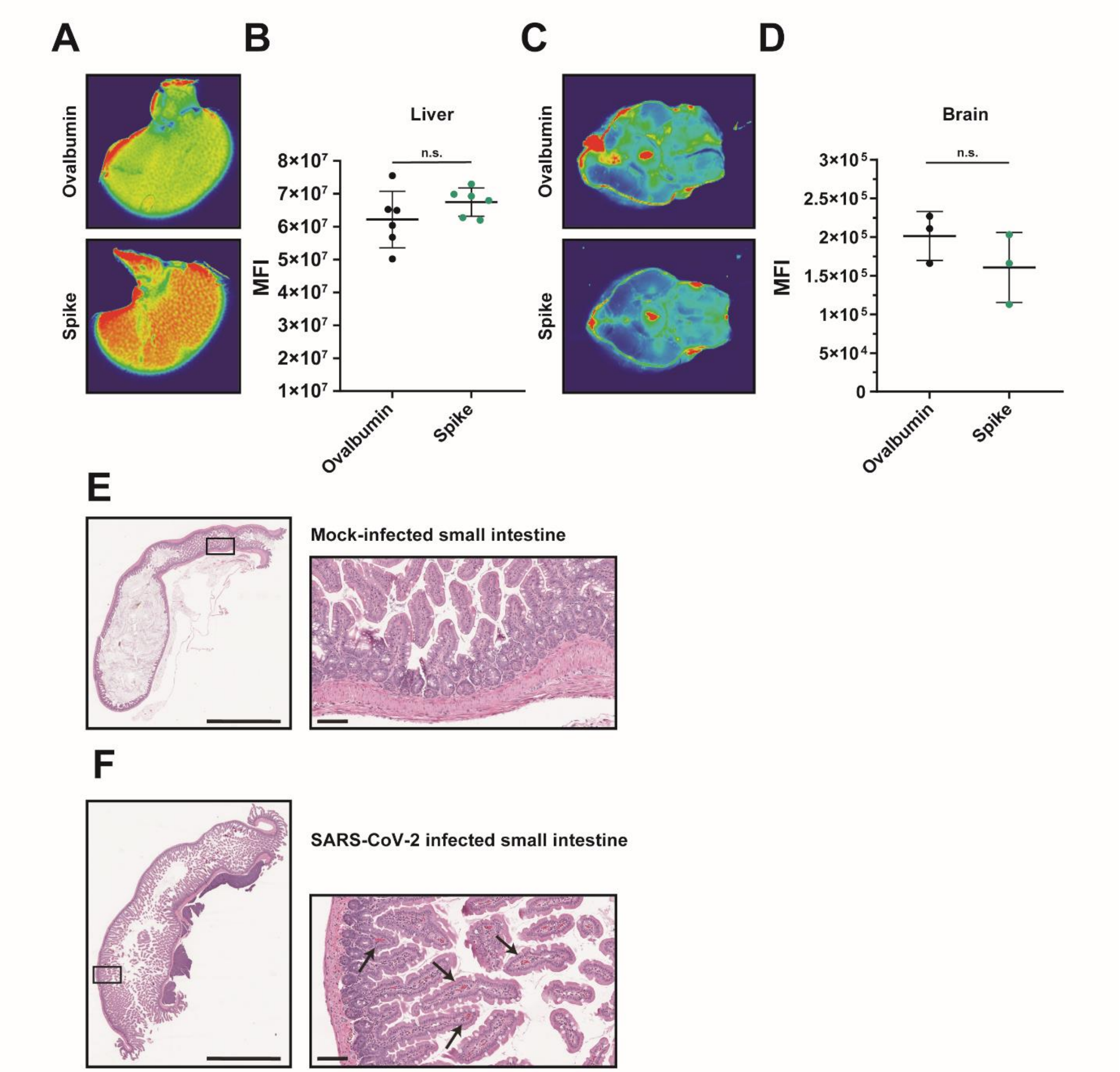

Figure S3. SARS-CoV-2 S and SARS-CoV-2 infection trigger vascular leak *in vivo* (Related to Figure 3). (A) Representative liver images from a SARS-CoV-2 S systemic vascular leak assay. Mice were administered 50 µg of SARS-CoV-2 S or ovalbumin as indicated, and 24 hpt were administered dextran-680 intravenously as in Figure 3. Organs from mice were collected, and accumulation of dextran-680 was measured with a fluorescent scanner. **(B)** Quantification of A from n=6 mice. **(C)** Same as A except representative images of brains. **(D)** Quantification of C from n=3 mice. All data are plotted as mean +/- SEM with n.s. $p>0.05$ by two-sided unpaired t-test. **(E-F)** Hematoxylin and eosin (H&E) staining was performed on small intestine sections from K18-hACE2 mice 7 days post-infection with 100 $TCID_{50}$ units of SARS-CoV-2 WA/1 isolate. Displayed are representative images of small intestines from n=3 mice in mock-infected conditions **(E)** and from n=4 mice infected with SARS-CoV-2 **(F),** left panels with scale bars at 2 mm and right panels consisting of zoomed-in insets with scale bars at 100 µm. Arrows point to dispersed red blood cells. Source data are provided as a Source Data file. All data are plotted as mean +/- SEM.

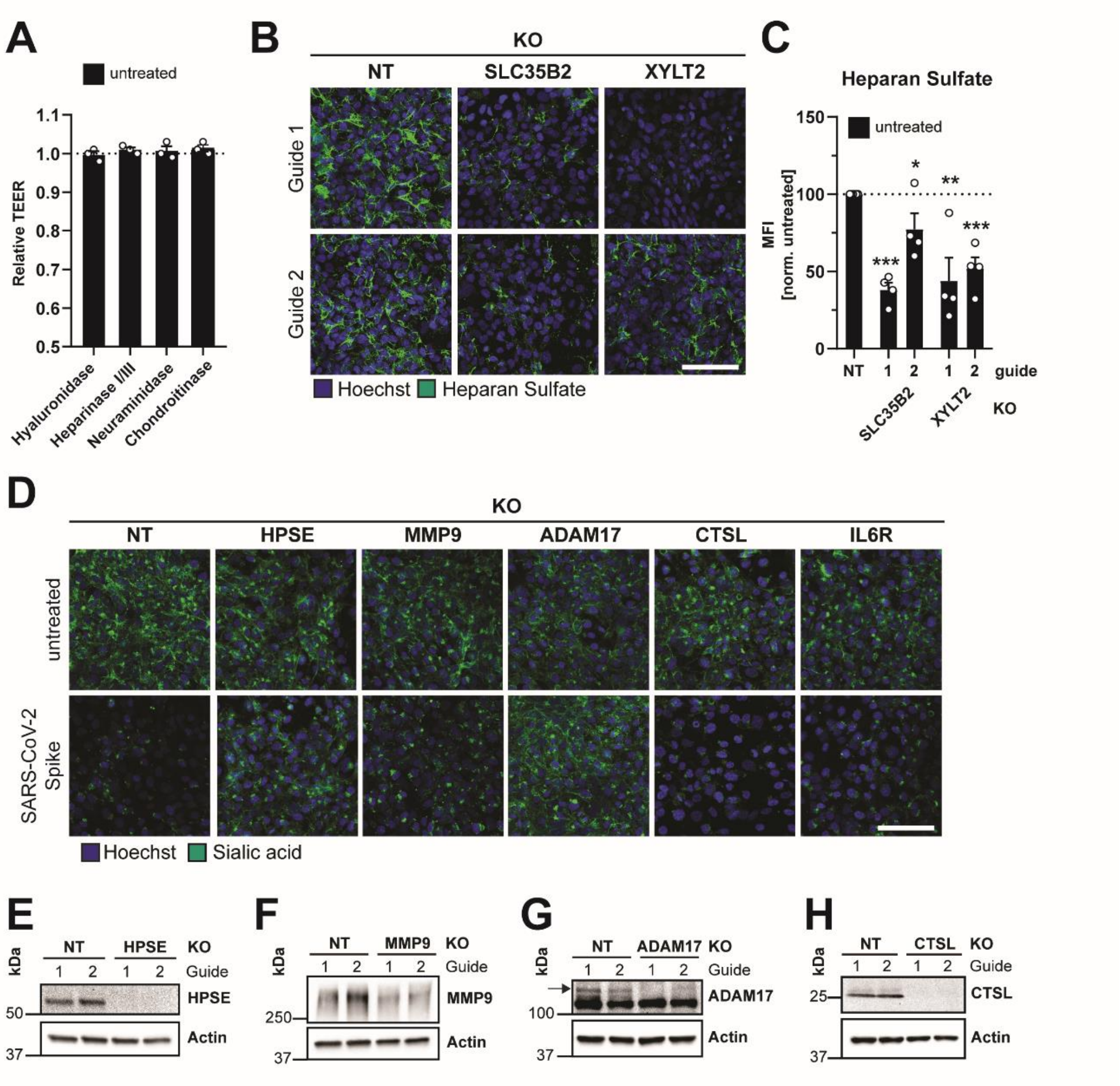

Figure S4. Glycans and EGL-modifying enzymes are required for S-mediated barrier dysfunction (Related to Figure 4). (A) TEER assay of HPMEC treated with recombinant hyaluronidase (10 µg/mL), heparin lyases I and III (5 mU/mL each), neuraminidase (1 U/mL), or chondroitinase (25 mU/mL). Data presented are control conditions from Figure 4B. Data are from n=3 biological replicates. **(B)** Representative IFA images of HPMEC transduced with lentivirus encoding the indicated guide RNAs and stained for heparan sulfate. Data presented are controls for Figure 4E. Displayed is one representative image from n=3 biological replicates. **(C)** Quantification of B from n=4 biological replicates. **(D)** A sialic acid EGL assay of HPMEC transduced with lentivirus encoding the indicated guide RNAs. HPMECs were treated with S (10 µg/mL) and stained 24 hours post-treatment. Data are representative IFA images from Figure 4G with n=3 biological replicates. **(E-H)** Western blot analyses for HPMECs from Figure 4G and Figure S4D probed for **(E)** heparanase (HPSE), **(F)** matrix metalloproteinase 9 (MMP9), **(G)** a disintegrin and metalloprotease 17 (ADAM17), and **(H)** Cathepsin L (CTSL). Actin was used as a loading control for all. All blots are from at least n=2 biological replicates. For all panels, heparan sulfate or sialic acid are stained in green and nuclei are stained with Hoechst in blue with scale

bars at 50 µm. MFI is mean fluorescence intensity. Dotted lines are the normalized untreated control conditions. All data are plotted as mean +/- SEM with $*p<0.05$, $**p<0.01$, $***p<0.001$, and n.s. $p>0.05$ by One-Way ANOVA with Tukey's Multiple comparisons test except for (C) which was analyzed by two-sided unpaired t-test compared to untreated controls. Source data are provided as a Source Data file.

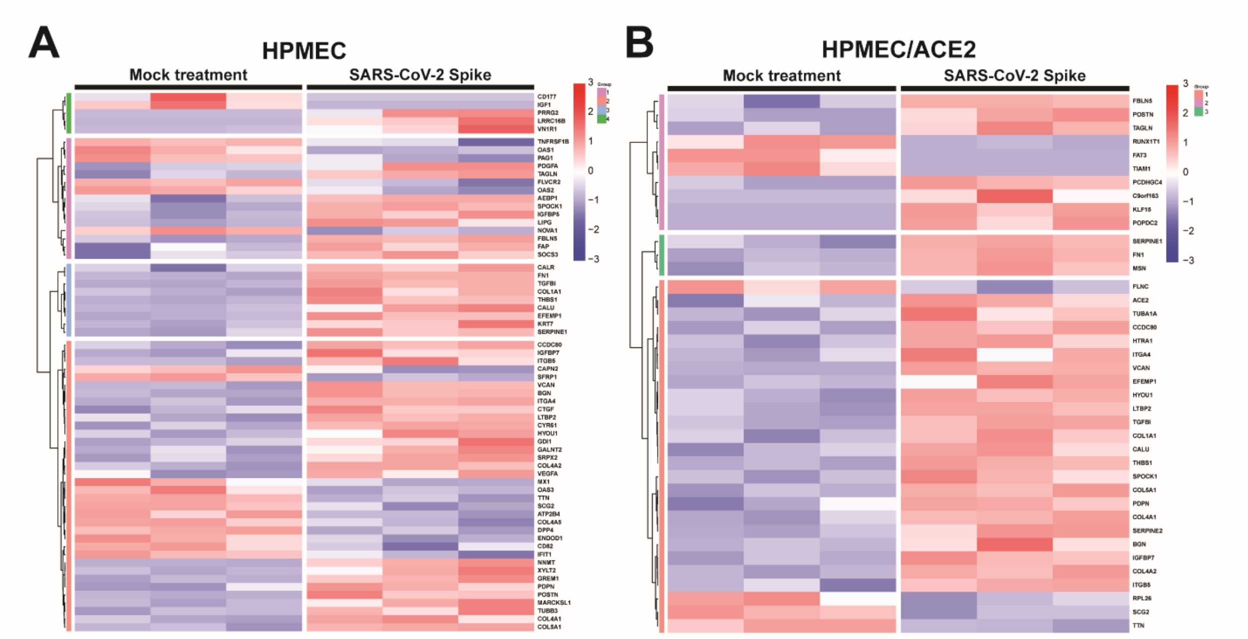

Figure S5. RNA-Seq of HPMEC and HPMEC/ACE2 treated with SARS-CoV-2 S (Related to Figure 5). (A) Heat map of DEGs identified in HPMECs treated with 10 µg/mL S for 24 hours. **(B)** Same as A except HPMEC/ACE2. The group designation refers to the cluster where the gene belongs after performing unsupervised hierarchical clustering. The color scale represents the z-score of normalized gene expression values. Statistical significance of DEGs was determined using a Wald test and a Benjamini-Hochberg (BH) p-value adjustment. DEGs with BH-corrected p-value <0.05 were included.

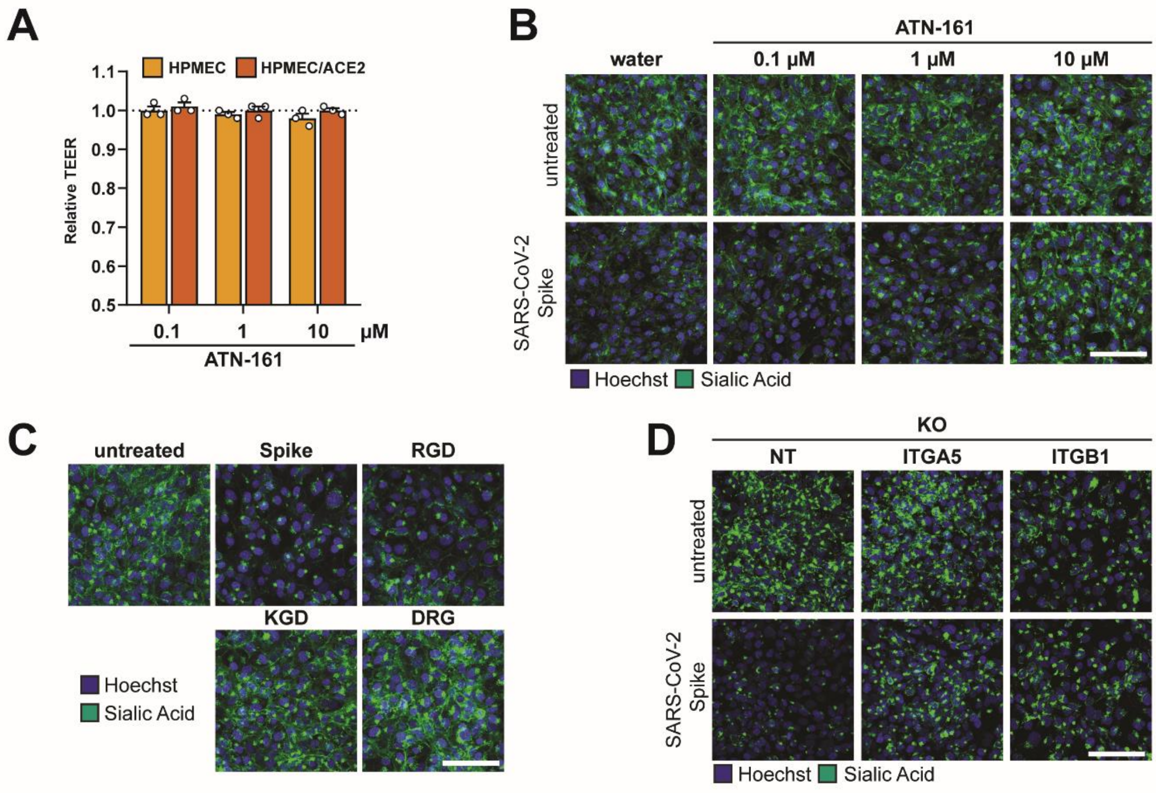

Figure S6. Integrins are required for SARS-CoV-2 S-mediated barrier dysfunction. (Related to Figure 6). (A) TEER assay of HPMEC treated with the indicated concentration of the integrin inhibitor ATN-161 for 24 hours. Dotted line is the normalized untreated control condition. These data are controls from Figure 6A and are from n=3 biological replicates. **(B)** Sialic acid EGL assay of HPMEC treated with 10 µg/mL of S and the indicated concentration of ATN-161. EGL was visualized 24 hpt. Data are representative images from Figure 6B and from at least n=3 biological replicates. **(C)** Sialic acid EGL assay as in B but treated with the indicated small peptides at 0.4 µM or S at 10 µg/mL. Data are representative images from Figure 6F from at least n=3 biological replicates. **(D)** Sialic acid EGL assay as in B but with the indicated CRISPR KO HPMECs. Data are representative images from Figure 6K with n=3 biological replicates. For all panels, sialic acid is stained in green and nuclei are stained with Hoechst in blue with scale bars at 50 µm. All data are plotted as mean +/- SEM. Source data are provided as a Source Data file.

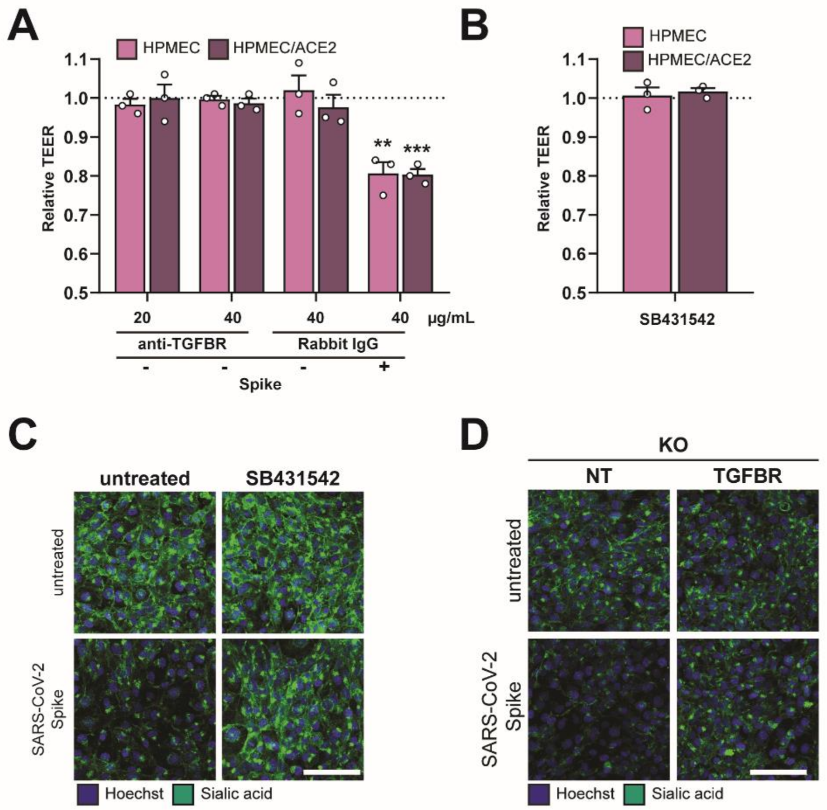

Figure S7. TGF-β signaling is required for SARS-CoV-2 S-mediated barrier dysfunction. (Related to Figure 7). (A) TEER assay of HPMEC treated with the indicated antibody and 10 µg/mL SARS-CoV-2 S. Data are control conditions from Figure 7C from n=3 biological replicates. **(B)** Same as A but treated with TGFBR inhibitor SB431542 (1 µM). Data are control conditions from Figure 7D from n=3 biological replicates. **(C)** Data are representative images from Figure 7E from n=3 biological replicates. **(D)** Data are representative images from Figure 7J from n=3 biological replicates. For all panels, sialic acid is stained in green and nuclei are stained with Hoechst in blue with scale bars at 50 µm. Dotted lines are the normalized untreated control conditions. All data are plotted as mean +/- SEM with **p<0.01 and ***p<0.001 by One-Way ANOVA with Tukey's Multiple comparisons test compared to untreated controls. Source data are provided as a Source Data file.

Supplementary Tables

Table S1: Table of all DEGs identified by RNA-seq for HPMEC. logFC is log2 fold change between S-treated and untreated HPMEC; lfcSE is standard error of logFC measurement; padj is the Benjamini-Hochberg adjusted p-value. DEGs sorted by padj in ascending order. Statistical significance of DEGs was determined using a Wald test and a Benjamini-Hochberg (BH) p-value adjustment. DEGs with BH-corrected p-value <0.05 were included.

	**logFC**	**lfcSE**	**padj**
TGFBI	1.371118	0.079699	3.56E-62
FN1	0.953106	0.066069	2.54E-43
VCAN	0.952346	0.087644	7.96E-24
COL5A1	1.26353	0.124811	1.55E-20
POSTN	1.383791	0.154461	9.37E-16
TAGLN	1.961064	0.226242	1.05E-14
THBS1	0.63853	0.073949	1.20E-14
BGN	0.647704	0.077844	1.56E-13
TTN	-0.90536	0.109385	2.01E-13
FBLN5	2.729718	0.334751	5.01E-13
COL4A2	0.773955	0.10208	4.42E-11
SPOCK1	1.286272	0.170702	5.80E-11
CCDC80	0.739803	0.098474	6.36E-11
ITGA4	0.594026	0.08376	1.35E-09
LTBP2	0.679898	0.098004	3.80E-09
EFEMP1	0.475991	0.069675	7.50E-09
COL4A1	0.817672	0.135217	1.24E-06
SERPINE1	0.409463	0.069775	3.49E-06
SCG2	-0.60211	0.103482	4.46E-06
GREM1	0.654721	0.117933	2.02E-05
COL1A1	0.431159	0.078372	2.56E-05
NNMT	0.600157	0.111027	4.20E-05
AEBP1	0.930373	0.176007	7.76E-05
PRRG2	8.333765	1.641456	0.000228
CTGF	0.398347	0.081507	0.000569
DPP4	-0.48372	0.099028	0.000569
PDGFA	1.273166	0.261147	0.000575
IGFBP5	0.706101	0.145405	0.000611
MARCKSL1	0.701964	0.145904	0.000739
SFRP1	-0.38668	0.081889	0.001112
TUBB3	0.6127	0.13006	0.001136
MX1	-0.76726	0.163872	0.001267
LIPG	0.612031	0.134572	0.002311
CYR61	0.366945	0.080743	0.002311

IGFBP7	0.464181	0.104312	0.003505
IFIT1	-0.55868	0.127617	0.004756
OAS1	-0.79358	0.183505	0.005898
PDPN	0.483369	0.112926	0.00701
CALR	0.325416	0.076226	0.007127
SRPX2	0.446491	0.10468	0.007127
OAS3	-0.51575	0.122883	0.009417
OAS2	-0.66492	0.162087	0.013912
PAG1	-0.71465	0.174574	0.0141
VN1R1	6.9542	1.701353	0.014154
CALU	0.41333	0.102276	0.016868
KRT7	0.338055	0.084121	0.01817
HYOU1	0.412395	0.102844	0.018456
LRRC16B	7.396787	1.854452	0.019768
XYLT2	0.503855	0.126489	0.019799
TNFRSF1B	-0.91591	0.230701	0.020517
COL4A5	-0.37191	0.093873	0.02083
ITGB5	0.361329	0.09158	0.021868
SOCS3	1.176033	0.301821	0.025852
FLVCR2	-0.53739	0.13793	0.025852
FAP	1.371427	0.357547	0.032514
ATP2B4	-0.34275	0.089574	0.033158
NOVA1	-1.09926	0.28812	0.034076
CAPN2	-0.38218	0.1007	0.035531
VEGFA	0.511021	0.134666	0.035531
CD82	-0.56799	0.149776	0.035531
GDI1	0.416437	0.110753	0.039769
ENDOD1	-0.37823	0.100823	0.040494
IGF1	-7.02501	1.881955	0.042918
CD177	-7.16087	1.922983	0.043724
GALNT2	0.416722	0.112014	0.043724

Table S2: Table of all DEGs identified by RNA-seq for HPMEC/ACE2. logFC is log2 fold change between S-treated and untreated HPMEC; lfcSE is standard error of logFC measurement; padj is the Benjamini-Hochberg adjusted p-value. DEGs sorted by padj in ascending order. Statistical significance of DEGs was determined using a Wald test and a Benjamini-Hochberg (BH) p-value adjustment. DEGs with BH-corrected p-value <0.05 were included.

	**logFC**	**lfcSE**	**padj**
FN1	1.155469	0.084239	1.11E-38
VCAN	1.365061	0.10998	1.55E-31
TGFBI	1.170262	0.104192	1.31E-25
ARNT2	20.72869	2.82404	5.88E-10
THBS1	0.677006	0.091917	5.88E-10
LTBP2	0.700158	0.105644	7.83E-08
FBLN5	3.224537	0.498445	1.94E-07
COL4A2	0.849703	0.132168	2.21E-07
TTN	-0.86289	0.137856	5.91E-07
TAGLN	2.00945	0.330354	1.63E-06
POSTN	1.694383	0.281304	2.14E-06
COL4A1	1.188305	0.198676	2.54E-06
TUBA1A	0.843212	0.14226	3.26E-06
COL5A1	0.965757	0.168631	1.00E-05
MSN	0.387547	0.073553	0.000126
SCG2	-0.6782	0.132551	0.000268
CALU	0.527747	0.103945	0.00031
CCDC80	0.590436	0.11718	0.000358
COL1A1	0.525848	0.104801	0.000379
SPOCK1	1.138718	0.231532	0.000599
HYOU1	0.511391	0.104165	0.000599
IGFBP7	0.679211	0.141543	0.000999
ACE2	0.49024	0.103388	0.001268
POPDC2	8.229141	1.764277	0.001776
ITGB5	0.646374	0.138927	0.001804
SERPINE1	0.365501	0.080207	0.002747
BGN	0.757858	0.16736	0.003031
FLNC	-0.47501	0.105658	0.003409
SERPINE2	0.818751	0.183007	0.003646
SCRG1	8.387214	1.960532	0.008651
PCDHGC4	4.979312	1.168255	0.008989
TIAM1	-7.87209	1.861754	0.010127
KLF15	7.792539	1.914317	0.019003
FAT3	-7.76917	1.908722	0.019003
C9orf163	7.877015	1.940926	0.019433
ITGA4	0.58027	0.145971	0.02688
EFEMP1	0.442293	0.112161	0.02948

RPL26	-0.566	0.143645	0.02948
RUNX1T1	-5.25768	1.340464	0.030956
PDPN	0.762492	0.194708	0.03097
HTRA1	0.42207	0.110751	0.046464

Table S3: Primers used to clone guide RNA sequences into the lentiCRISPR v2 plasmid**.**

Target	Guide RNA Sequence
gRNA_NTG_1_F	CACCGTACTAACGCCGCTCCTACAG
gRNA_NTG_1_R	AAACCTGTAGGAGCGGCGTTAGTAC
gRNA_NTG_2_F	CACCGGATCCAGGAGTGATCGAGTA
gRNA_NTG_2_R	AAACTACTCGATCACTCCTGGATCC
gRNA_ACE2_59272_1_F	CACCGAACATCTTCATGCCTATGTG
gRNA_ACE2_59272_1_R	AAACCACATAGGCATGAAGATGTTC
gRNA_ACE2_59272_2_F	CACCGCAGGATCCTTATGTGCACAA
gRNA_ACE2_59272_2_R	AAACTTGTGCACATAAGGATCCTGC
gRNA_CTSL_1514_1_F	CACCGAGATGTTCCGGAAAACTGGG
gRNA_CTSL_1514_1_R	AAACCCCAGTTTTCCGGAACATCTC
gRNA_CTSL_1514_2_F	CACCGCAGTATGTTCAGGATAATGG
gRNA_CTSL_1514_2_R	AAACCCATTATCCTGAACATACTGC
gRNA_HPSE_10855_1_F	CACCGTAAAAATGTCCAATACATCA
gRNA_HPSE_10855_1_R	AAACTGATGTATTGGACATTTTTAC
gRNA_HPSE_10855_2_F	CACCGTGGCAATCTCAAGTCAACCA
gRNA_HPSE_10855_2_R	AAACTGGTTGACTTGAGATTGCCAC
gRNA_TGFBR1_7046_1_F	CACCGAGAACGTTCGTGGTTCCGTG
gRNA_TGFBR1_7046_1_R	AAACCACGGAACCACGAACGTTCTC
gRNA_TGFBR1_7046_2_F	CACCGATGGGCAAGACCGCTCGCCG
gRNA_TGFBR1_7046_2_R	AAACCGGCGAGCGGTCTTGCCCATC
gRNA_ADAM17_6868_1_F	CACCGAATCAGAATCAACACAGATG
gRNA_ADAM17_6868_1_R	AAACCATCTGTGTTGATTCTGATTC
gRNA_ADAM17_6868_2_F	CACCGACAAAATTTCAAGGTCGTGG
gRNA_ADAM17_6868_2_R	AAACCCACGACCTTGAAATTTTGTC
gRNA_IL6R_3570_1_F	CACCGCCGTGGCCAGAAACCCCCGC
gRNA_IL6R_3570_1_R	AAACGCGGGGGTTTCTGGCCACGGC
gRNA_IL6R_3570_2_F	CACCGTGGAAACTATTCATGCTACC
gRNA_IL6R_3570_2_R	AAACGGTAGCATGAATAGTTTCCAC
gRNA_MMP9_4318_1_F	CACCGACTACTCGGAAGACTTGCCG
gRNA_MMP9_4318_1_R	AAACCGGCAAGTCTTCCGAGTAGTC
gRNA_MMP9_4318_2_F	CACCGCCGCTATGGTTACACTCGGG
gRNA_MMP9_4318_2_R	AAACCCCGAGTGTAACCATAGCGGC
gRNA_XYLT2_64132_1_F	CACCGAGGACACAGACAGTTCAGCA
gRNA_XYLT2_64132_1_R	AAACTGCTGAACTGTCTGTGTCCTC
gRNA_XYLT2_64132_2_F	CACCGCCAGGGCTATGATAACGTGC
gRNA_XYLT2_64132_2_R	AAACGCACGTTATCATAGCCCTGGC
gRNA_SLC35B2_347734_1_F	CACCGCAGGTGTCTTATCTGACTTG

gRNA_SLC35B2_347734_1_R	AAACCAAGTCAGATAAGACACCTGC
gRNA_SLC35B2_347734_2_F	CACCGCTGGGTCCATGACTCCGGAG
gRNA_SLC35B2_347734_2_R	AAACCTCCGGAGTCATGGACCCAGC
gRNA_ITGA5_3678_1_F	CACCGCCCCGAGTACCTGATCAACC
gRNA_ITGA5_3678_1_R	AAACGGTTGATCAGGTACTCGGGGC
gRNA_ITGA5_3678_2_F	CACCGTGGATCGGACCCCTGACGGG
gRNA_ITGA5_3678_2_R	AAACCCCGTCAGGGGTCCGATCCAC
gRNA_ITGB1_3688_1_F	CACCGAATGTAACCAACCGTAGCAA
gRNA_ITGB1_3688_1_R	AAACTTGCTACGGTTGGTTACATTC
gRNA_ITGB1_3688_2_F	CACCGGAACGGGGTGAATGGAACAG
gRNA_ITGB1_3688_2_R	AAACCTGTTCCATTCACCCCGTTCC
