# Supplementary material for: SARS-CoV-2 Spike triggers barrier dysfunction and vascular leak via integrins and TGF-β signaling
Source: Nat Commun. 2022 Dec 9;13:7630. doi: 10.1038/s41467-022-34910-5 (PMC9734751; doi:10.1038/s41467-022-34910-5)
Supplement: Supplementary file 3 — Reporting Summary [file 41467_2022_34910_MOESM3_ESM.pdf]

nature portfolio

Corresponding author(s): Eva Harris, Scott Biering

Last updated by author(s): November 3rd, 2022

Reporting Summary

Nature Portfolio wishes to improve the reproducibility of the work that we publish. This form provides structure for consistency and transparency in reporting. For further information on Nature Portfolio policies, see our Editorial Policies and the Editorial Policy Checklist.

Statistics

For all statistical analyses, confirm that the following items are present in the figure legend, table legend, main text, or Methods section.

n/a	Confirmed	
☐	☒	The exact sample size (n) for each experimental group/condition, given as a discrete number and unit of measurement
☐	☒	A statement on whether measurements were taken from distinct samples or whether the same sample was measured repeatedly
☐	☒	The statistical test(s) used AND whether they are one- or two-sided<br>*Only common tests should be described solely by name; describe more complex techniques in the Methods section.*
☐	☒	A description of all covariates tested
☐	☒	A description of any assumptions or corrections, such as tests of normality and adjustment for multiple comparisons
☐	☒	A full description of the statistical parameters including central tendency (e.g. means) or other basic estimates (e.g. regression coefficient) AND variation (e.g. standard deviation) or associated estimates of uncertainty (e.g. confidence intervals)
☐	☒	For null hypothesis testing, the test statistic (e.g. F, t, r) with confidence intervals, effect sizes, degrees of freedom and P value noted<br>*Give P values as exact values whenever suitable.*
☒	☐	For Bayesian analysis, information on the choice of priors and Markov chain Monte Carlo settings
☒	☐	For hierarchical and complex designs, identification of the appropriate level for tests and full reporting of outcomes
☒	☐	Estimates of effect sizes (e.g. Cohen's d, Pearson's r), indicating how they were calculated

Our web collection on statistics for biologists contains articles on many of the points above.

Software and code

Policy information about availability of computer code

Data collection	No software was used for data collection.
Data analysis	All data were plotted and quantitative analyses performed using GraphPad Prism 8 software. All western blot images were captured on a ChemiDoc system with Image Lab software version 6.01 (Bio-Rad). All in vivo vascular leak assays were analyzed on a fluorescence scanner (LI-COR Odyssey CLx Imaging system) and analyzed with Image Studio Lite software version 5.2.

For manuscripts utilizing custom algorithms or software that are central to the research but not yet described in published literature, software must be made available to editors and reviewers. We strongly encourage code deposition in a community repository (e.g. GitHub). See the Nature Portfolio guidelines for submitting code & software for further information.

Data

Policy information about availability of data

All manuscripts must include a data availability statement. This statement should provide the following information, where applicable:

- Accession codes, unique identifiers, or web links for publicly available datasets
- A description of any restrictions on data availability
- For clinical datasets or third party data, please ensure that the statement adheres to our policy

All raw data associated with this study are provided as as source data file. Analyzed RNA-Seq data are included in the manuscript as supplementary tables and raw data are uploaded to NCBI SRA at BioProject accession # PRJNA807823.

Field-specific reporting

Please select the one below that is the best fit for your research. If you are not sure, read the appropriate sections before making your selection.

- [x] Life sciences
- [ ] Behavioural & social sciences
- [ ] Ecological, evolutionary & environmental sciences

For a reference copy of the document with all sections, see nature.com/documents/nr-reporting-summary-flat.pdf

Life sciences study design

All studies must disclose on these points even when the disclosure is negative.

Sample size	No sample size calculation was conducted. We selected samples sizes of sufficient size to ensure reproducibility of our findings as well as large enough to perform statistical analyses (at least n=3 unless stated otherwise). Our rational for selecting this sample size was primarily based on previous experience with these assays informing us on the number of replicates required to achieve statistical significance given the variance of each assay.
Data exclusions	No data were excluded from this study. We pre-established our exclusion criteria as a rejection of a dataset if either our positive controls or negative controls failed.
Replication	We repeated experiments at least three times or the number indicated in the figures legends.
Randomization	Experiments were not randomized. Data variability was controlled through the inclusion of multiple biological replicates, inclusion of multiple technical replicates within an experiment, and utilization of distinct guide RNAs targeting a single gene in multiple cell lines.
Blinding	Researchers were not blinded during experiments which was a result of working with infectious SARS-CoV-2 and the high complexity of the conducted experiments.

Reporting for specific materials, systems and methods

We require information from authors about some types of materials, experimental systems and methods used in many studies. Here, indicate whether each material, system or method listed is relevant to your study. If you are not sure if a list item applies to your research, read the appropriate section before selecting a response.

Materials & experimental systems

n/a	Involved in the study	
☐	☒	Antibodies
☐	☒	Eukaryotic cell lines
☒	☐	Palaeontology and archaeology
☐	☒	Animals and other organisms
☒	☐	Human research participants
☒	☐	Clinical data
☒	☐	Dual use research of concern

Methods

n/a	Involved in the study	
☒	☐	ChIP-seq
☒	☐	Flow cytometry
☒	☐	MRI-based neuroimaging

Antibodies

Antibodies used	The following antibodies were used in this study: goat anti-ACE2 (R&D Systems, AF933), rabbit anti-integrin alpha 5 (Abcam, ab150361), rabbit anti-ITGB1 (Thermo Fisher Scientific, PA5-29606), rabbit anti-Heparanase 1 (Abcam, EPR22365-230, ab254254), rabbit anti-MMP-9 (Cell Signaling Technology, #3852), mouse anti-TACE/ADAM17 (Santa Cruz Biotechnologies, B-6, sc-390859), mouse anti-Cathepsin L (Thermo Fisher Scientific, 33-2, BMS1032), rabbit anti-TGFBR1 (Thermo Fisher Scientific, PA5-32631), mouse anti-His (MA1-21315, Thermo Scientific), mouse anti-β-actin HRP (Santa Cruz Biotechnologies, sc-47778 HRP), goat anti-mouse HRP (Biolegend, 405306), donkey anti-rabbit HRP (Biolegend, 406401), donkey anti-human HRP, Biolegend, 410902), hyaluronic acid (Abcam ab53842), heparan sulfate (amsbio, clone F58-10E6, 370255-s), or chondroitin sulfate (Thermo Fisher Scientific, clone CS-56, ma1-83055), hyaluronidase (Abcam, clone PH20, ab196596) and neuraminidase 2 (Thermo Fisher Scientific, pa5-35114), anti-Spike (Genetex, 1A9, GTX632604), anti-Spike (Absolute Antibody, CR3022), rabbit anti-TGFBR1 (Thermo Fisher Scientific, PA5-32631). All primary antibodies were used at a dilution of 1:1000 and all secondary antibodies were used at a concentration of 1:5000.
Validation	Each antibody was validated using cellular lysates from both positive and negative controls (parental vs. KO cells). Only antibodies that validated according to the manufactures instructions were used in this study.

Eukaryotic cell lines

Policy information about cell lines

Cell line source(s)	HEK293T, Calu-3, and Vero cells were obtained from ATCC via the UC Berkeley cell culture facility. Human pulmonary

	microvascular endothelial cells (HPMEC) [line HpMEC-ST1.6R] were a gift from Dr. J.C. Kirkpatrick at Johannes Gutenberg University, Germany.
Authentication	Cells came authenticated from ATCC. HPMEC are routinely screened by IFA for endothelial cell specific markers (including VE-Cadherin) while Calu-3 were screened by IFA for epithelial cell specific markers (including E-Cadherin).
Mycoplasma contamination	Cells were certified mycoplasma free from the UC Berkeley Cell Culture Facility/Lonza upon receipt. Cell in the Harris lab are routinly screened for mycoplasma.
Commonly misidentified lines (See ICLAC register)	HEK293T

Animals and other organisms

Policy information about studies involving animals; ARRIVE guidelines recommended for reporting animal research

Laboratory animals	Six- to eight-week-old wild-type C57BL/6J and K18-hACE2 [B6.Cg-Tg(K18-ACE2)2Prlmn/J] mice of both genders were purchased from the Jackson Laboratory and housed at the University of California, Berkeley Animal Facility under specific pathogen-free conditions. Mice were housed in temperature-controlled environments on a 12-hour light and dark cycle, with food and water provided ad libitum. All experiments and procedures were pre-approved by the UC Berkeley Animal Care and Use Committee, Protocol AUP-2014-08-6638-2 and AUP-2020-07-13458 and conducted in compliance with Federal and University regulations. We used female mice for all vascular leak assays and male mice for all SARS-CoV-2 infections.
Wild animals	No wild animals were used in this study.
Field-collected samples	No field-collected samples were used in this study.
Ethics oversight	Mice were housed at the UC Berkeley Animal Facility in accordance with all state and federal regulations. All animal work was approved by Institutional Animal Care and Use Committee at the University of California, Berkeley.

Note that full information on the approval of the study protocol must also be provided in the manuscript.
