# Supplementary figures and images for: SARS-CoV-2 Spike triggers barrier dysfunction and vascular leak via integrins and TGF-β signaling
Source: Nat Commun. 2022 Dec 9;13:7630. doi: 10.1038/s41467-022-34910-5 (PMC9734751; doi:10.1038/s41467-022-34910-5)

**Figure 6I**

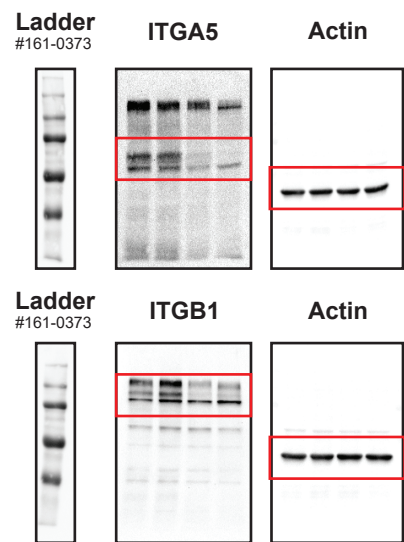

**Figure 7H**

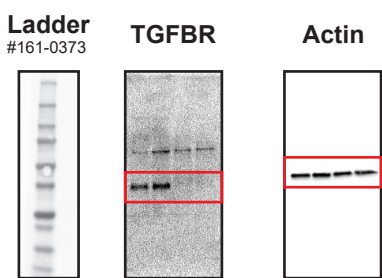

**Supplementary Figure 1A**

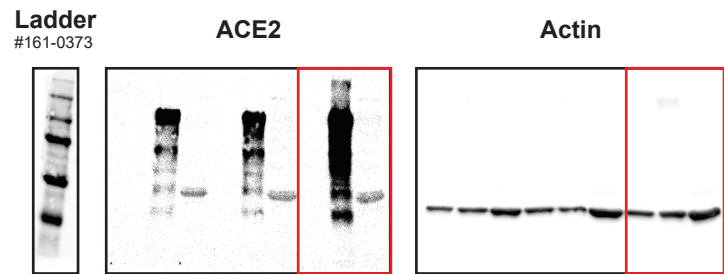

**Supplementary Figures 4E, F, G, H**

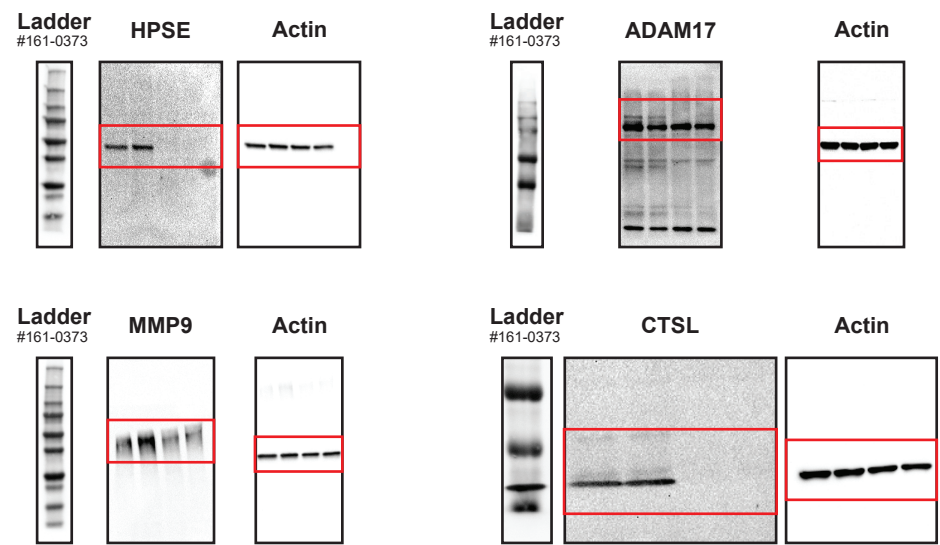

Supplement: Supplementary file 4 — source data [file 41467_2022_34910_MOESM4_ESM.zip › WB_raw.pdf]
